# Complementary and Integrative Health Practices Among Hispanics Diagnosed with Colorectal Cancer: Utilization and Communication with Physicians

**DOI:** 10.1089/acm.2015.0332

**Published:** 2016-06-01

**Authors:** David S. Black, Chun Nok Lam, Nathalie T. Nguyen, Ugonna Ihenacho, Jane C. Figueiredo

**Affiliations:** ^1^ Department of Preventive Medicine, Keck School of Medicine of University of Southern California, Los Angeles, CA.; ^2^ Norris Comprehensive Cancer Center, Keck School of Medicine of University of Southern California, Los Angeles, CA.; ^3^ Department of Emergency Medicine, Keck School of Medicine of University of Southern California, Los Angeles, CA.

## Abstract

***Objective:*** Complementary and integrative health (CIH) use among Hispanic adults with colorectal cancer (CRC) diagnosis is not well documented. Understanding the prevalence and patterns of CIH use among Hispanics offers insights to uncover potential needs for clinical services.

***Design:*** Participants were age 21 years or older with a first-time diagnosis of CRC from population-based cancer registries in California. In-person and/or telephone-based interviews were administered to collect data on CIH use. Demographic and clinical diagnosis data were abstracted from medical records. Descriptive statistical and logistic regression was used to analyze the frequencies and associations between selected patient characteristics and CIH use.

***Results:*** Among 631 Hispanic patients, 40.1% reported ever using CIH. Herbal products/dietary supplements were used most often (35.3%), followed by bodywork (16.5%), mind–body practices (7.8%), and homeopathy (6.7%). About 60% of participants reported CIH use to address specific health conditions; however, most patients did not discuss CIH use with their physicians (76.3%). Women reported higher CIH use than did men (45.1% versus 35.9%; odds ratio, 1.49 [95% confidence interval, 1.07–2.08]; *p* = 0.02). CIH use did not differ by clinical stage, time since diagnosis, or preferred language.

***Conclusions:*** CIH use is prevalent among Hispanic patients with CRC, especially women. Little communication about CIH use occurs between participants and their healthcare providers. Efforts aimed at improving integrative oncology services provide an opportunity to address such gaps in healthcare service.

## Introduction

Complementary and integrative health (CIH) approaches refer to the array of products and techniques that are used by the public outside of or alongside conventional Western medicine. According to the most recent report of the National Institutes of Health (NIH), 34% of adults in the United States use CIH practices.^1^ Overall, this rate of use has remained stable over the past decade, while some CIH mind–body practices, such as meditation and yoga, have almost doubled. The U.S. public does not limit itself to conventional Western medicine and, further, does not see conventional medicine and CIH as mutually exclusive practices to bringing them closer to their goal for health and wellness.^2,3^ Therefore, CIH has major implications for U.S. public health, as users are more likely than nonusers to rate their own health as excellent and their health as better than the previous year.^4^

Several mind–body CIH approaches have an emerging evidence base suggesting their utility for ameliorating health ailments,^5–12^ and some natural products have also shown promise;^13–18^ however, methodologic and conceptual limitations do prevail in various research domains of CIH,^18,19^ and null findings are noted for some products.^20,21^ Certain segments of the population report higher than the national average of CIH use. Rates of CIH use among individuals with chronic disease increase to over half of the population.^22^ This is exemplified in the population of more than 12 million people living with a cancer diagnosis, of whom 40%–80% report CIH use.^23,24^ Prevalence appears to increase after a cancer diagnosis and in instances of advanced cancer stage,^25–27^ perhaps as patients and families attempt to remediate lingering iatrogenic symptoms and improve their quality of life by less mainstream approaches. Breast cancer in women has served as the central research model for understanding CIH use and efficacy in the context of cancer, while research on other cancer types remains a relatively nascent area. Notwithstanding this limitation of cancer type and sex, integrative oncology is rapidly gaining ground in the United States,^28^ and clinical guidelines on the use of CIH approaches for patients with cancer have been provided for healthcare practitioners by the Society for Integrative Oncology.^29,30^

It is well known that socioeconomic status, language, and ethnicity can act as barriers to medical care access.^31–33^ Spanish-language preference marks a particularly vulnerable subpopulation in the United States that has less access to care^34^ and is becoming a majority in several urban regions. Notable population-based differences in CIH use occur by ethnicity and socioeconomic status, wherein Hispanics report lower CIH use than whites.^35^ Various explanations include acculturation factors and lack of access to care.^36–39^ However, this might also reflect underreporting bias given that various CIH practices for the treatment of illness and disease are embedded within a Hispanic cultural norm that does not expound on CIH use.^39^ More recently, Hispanics show an increased use of certain CIH mind–body practices, especially yoga, which has almost doubled from 2.7% to 5.1% of the population from 2007 to 2012 (https://nccih.nih.gov/research/statistics/NHIS).

Relatively little is known about CIH use in Hispanics with a diagnosis of colorectal cancer (CRC). Existing data from several international studies show that CIH use among patients with CRC ranges in prevalence between 20% and 50%;^27,40–42^ however, the two U.S. studies suggest inconsistent findings, with use at 31% and 75%.^43,44^ To better understand the prevalence and patterns of CIH use among Hispanic patients with CRC living in the United States, this large population-based study in California sought to describe the CIH products and techniques used by Hispanic adults. The goal of this research is to advance knowledge on the use of CIH practices in diverse U.S. populations in order to inform best healthcare practices to be used with Hispanic cancer survivors.

## Materials and Methods

### Participants and procedures

Data were used from participants in the Hispanic Colorectal Cancer Study, which is a population-based study of individuals self-identified as Hispanic or Latino with a diagnosis of CRC. All men and women older than 21 years of age with a first time diagnosis of CRC (International Classification of Diseases for Oncology, Third Edition, codes: C18–C20) after January 2008 were eligible for participation. Cases were identified from the California Cancer Registry and/or from hospitals in Los Angeles (LAC+USC County Hospital and USC Norris Comprehensive Cancer Center). As of September 2015, 1641 patients were recruited, of whom 633 (39%) completed the psychosocial questionnaires, and 2 (0.3%) patients were excluded from analysis because of missing data. All patients were post–first-line treatment and 70% had survived at least 3 years. The University of Southern California Institutional Review Board approved all study protocols.

### Measures

All patients were interviewed in person or by telephone. Patient demographic information was obtained using a standardized risk factor questionnaire from the Colon Cancer Family Registry.^19^ The eight items measuring CIH use in the past 12 months were taken directly from the 2011 NIH National Center for Complementary and Integrative Health survey in an effort to compare prevalence estimates from the probability sample and recent national prevalence reports published by the NIH. Cancer localization (local, regional, or distant) and first-line treatment data were obtained from the California Cancer Registry, a population-based cancer surveillance program that is part of the California Department of Public Health. Study data were collected and managed using REDCap electronic data capture tools hosted at the University of Southern California.^45^

### Statistical analysis

Demographic characteristics for the sample were summarized using means and frequencies. Chi-square tests and *t*-tests were used as appropriate to determine statistical significance in descriptive analyses. Logistic regression was used to determine the association between patient characteristics and use of CIH adjusted for potential confounders. A *p*-value <0.05 indicated a statistically significant difference. Mean values by sex were imputed for missing data on age at the time of diagnosis for the regression models. Analyses were performed with SAS software, version 9.4 (SAS Institute Inc., Cary, NC).

## Results

There were 631 (54.4% male) patients included in the analysis; the mean age at time of diagnosis was 56.9 years (standard deviation [SD], 12.2) (Table 1). Mean time from interview to diagnosis was 3.0 years (SD, 1.2) and did not differ by sex. Over half of the patients were diagnosed with CRC (57.2%) and had regional or localized disease (89.6%). Approximately half (50.2%) did not receive chemotherapy or radiation after diagnosis.

**Table 1.** Characteristics of Participants in the Hispanic Colorectal Cancer Study

*Variable*	*Value (*n* = 631)*
Age (yr)	59.5 ± 12.3
Age at diagnosis (yr)	56.9 ± 12.2
Missing	82
Sex	
Male	344 (54.4)
Female	287 (45.6)
Income	
<$15,000	118 (22.3)
$15,000–$29,000	152 (28.7)
$30,000–$44,000	82 (15.5)
$45,000–$69,000	81 (15.3)
≥$70,000	96 (18.2)
Missing	102
Preferred language	
English	296 (46.9)
Spanish	335 (53.1)
Cancer site	
Colon	314 (57.2)
Rectum	235 (42.8)
Missing	82
Treatment	
No chemotherapy/radiation	265 (50.2)
Chemotherapy only	135 (25.6)
Chemotherapy and radiation	124 (23.5)
Radiation only	4 (0.8)
Missing	103
Clinical stage	
Localized	211 (40.8)
Regional	252 (48.7)
Remote	54 (10.4)
Missing	114
Time since diagnosis	
≥3 yr	384 (70.0)
<3 yr	165 (30.1)
Missing	82
History of polyps	33 (53.8)
Don't know/missing	16
Reason for first colonoscopy	
Investigate new problem	366 (58.0)
Family history of CRC	20 (3.2)
Routine/yearly check-up	127 (20.1)
Follow-up previous problem	78 (12.4)
Follow-up of fecal blood test	38 (6.0)
Other	60 (9.5)

Values are expressed as *n* (%) or mean ± standard deviation.

CRC, colorectal cancer.

More than a third of the study population reported using at least one form of CIH (40.1%) (Table 2). Overall, herbal products and dietary supplements were the most commonly used CIH approach in the past 12 months (35.3%), followed by bodywork (16.5%), mind–body practices (7.8%), and homeopathy (6.7%). Among the CIH users, most reported use to target a specific health condition (59.3%), to prevent disease and promote overall wellness (59.8%), or to ameliorate painful conditions (43.4%). Only 23.7% of the patients had discussed CIH with their physicians. Some individuals reported discussing CIH use with a nurse or nurse practitioner (8.5%), pharmacist (2.1%), physician's assistant (4.5%), and other healthcare provider (2.2%) (data not shown). The most highly reported reason for not discussing CIH with a healthcare provider was lack of knowledge to report (25.6%). Overall, family or friends were the most frequent source of CIH information (26.9%), followed by radio/TV (7.7%) and the Internet (7.5%).

**Table 2.** Complementary and Integrative Health Use Among Participants in the Hispanic Colorectal Cancer Study

*Variable*	N = *631(%)*
Ever use of CIH	253 (40.1)
Past 12 mo use among users	
Herbal products and dietary supplements	222 (35.3)
Bodywork	104 (16.5)
Mind–body practices	49 (7.8)
Homeopathy	42 (6.7)
Other	24 (3.9)
Reasons for CIH use among users	
Specific health condition	147 (59.3)
Prevention/overall wellness	147 (59.8)
Painful conditions	108 (43.4)
Supplement conventional medicine	93 (37.7)
Other reason	14 (5.8)
Reasons CIH use was not discussed with health provider	
Didn't know you should	149 (25.6)
Healthcare provider never asked	142 (24.1)
Not enough time during office visit	72 (12.3)
Didn't think healthcare provider knows about the topic	55 (9.4)
Weren't comfortable discussing it with your healthcare provider	51 (8.7)
Healthcare provider would have been dismissive or told you not to do it	53 (9.1)
Other reason	88 (14.8)
Primary source of CIH information among users	
Family/friend	157 (26.9)
Internet	44 (7.5)
Radio/TV	45 (7.7)
Physician	34 (5.8)
Publications	10 (1.7)
Nutritionist	6 (1.0)
Physician assistant	2 (0.3)
Pharmacist	2 (0.3)
Health food stores	3 (0.5)
Other	6 (1.0)
No CIH information source	273 (46.8)

Proportions may vary slightly due to missing values.

CIH, complementary and integrative health.

In Table 3, sex was the only patient characteristic associated with CIH use after adjusting for other covariates (women, 45.1% ; men, 35.9%). Overall, women were nearly 50% more likely to have ever used one or more forms of CIH compared to men (multivariable-adjusted odds ratio, 1.49; 95% confidence interval, 1.07–2.08). Other patient characteristics, including age, cancer stage, time since diagnosis, treatment, and preferred language, were not associated with CIH use.

**Table 3.** Association Between Patient Characteristics and Ever Use of CIH

*Variable*	*Crude OR (95% CI)*	p*-Value*	*Adjusted OR (95% CI)^a^*	p*-Value*
Age at diagnosis^b^				
Age ≥50 yr	1		1	
Age <50 yr	1.32 (0.90–1.95)	0.16	1.14 (0.76–1.71)	0.53
Sex				
Male	1		1	
Female	1.47 (1.07–2.03)	0.02	1.49 (1.07–2.08)	0.02
Language				
English	1		1	
Spanish	0.70 (0.51–0.97)	0.03	0.90 (0.60–1.44)	0.60
Time since diagnosis				
≥3 yr	1		1	
<3 yr	1.05 (0.72–1.52)	0.80	1.03 (0.70–1.52)	0.87
Tumor localization				
Local	1		1	
Regional	0.73 (0.50–1.06)	0.10	0.75 (0.51–1.10)	0.14
Remote	1.29 (0.71–2.35)	0.40	1.16 (0.62–2.16)	0.64
Cancer treatment				
No chemotherapy or radiation	1		1	
Chemotherapy and/or radiation	0.89 (0.63–1.26)	0.53	0.87 (0.60–1.25)	0.45

^a^ Models adjusted for sex, age at diagnosis, income, preferred language, and education level.

^b^ Average age at diagnosis for men and average age at diagnosis for women imputed for missing values.

CI, confidence interval; OR, odds ratio.

## Discussion

To better understand the prevalence and patterns of CIH use among Hispanic patients with CRC living in the United States, this study assessed the CIH products and techniques used by Hispanic adults in a large population-based study in California. Results showed that CIH use is prevalent in this population, in lieu of possible underreporting bias in Hispanics.^39^ Women were more likely than men to use herbal products and dietary supplements, mind–body practices, and homeopathy in the past 12 months. Use of CIH was not dependent on clinical stage of cancer, time since diagnosis, or preferred language, suggesting that disease-specific and acculturation factors have minimal confounding effects on the explanation for CIH use in this population. Most CIH users reported using CIH to alleviate specific health conditions and for purposes of wellness, indicating active participation from survivors to enhance their quality of life during treatment and/or survivorship stages.^46^ The prevailing use of CIH in the Hispanic population suggests that the broader movement of integrative oncology care is in need for the less-studied minority populations.^29,30,47^

The prevalence of CIH use among Hispanic cancer survivors observed in this population-based study (41%) falls within the range (40%–80%) of patients living with cancer, and CRC specifically (20%–50%) when considering international data;^27,40–42^ it is higher than the national average of CIH among all adults (34%).^23,24^ When compared with the prevalence of CIH use among Hispanics living without cancer (50%–90%),^39,48^ the current findings suggested a lower rate of use. A possible explanation is that previous studies often measured CIH use with measurement indicators specific to the Hispanic population/culture and therefore were more sensitive to positive reporting. Although the NIH-based measurement of CIH use applied in this study may be less sensitive for capturing Hispanic-specific CIH use, the results still suggest that many Hispanic patients with cancer practice CIH alongside conventional care options as a goal to pursue health and wellness.^2,3^

Because few studies have focused on CIH use in minority populations with more aggressive forms of cancer, such as with the case of CRC, these results call for the further study of the safety and effectiveness of CIH products and services that are already used in the daily life of cancer patients and survivors from diverse race/ethnic backgrounds and cancer types.^49^ Our observation of higher CIH use among women than men also aligns with previous literature.^50–52^ Studies have suggested that women may have greater awareness and access to CIH resources because of their nurturing roles in the family and greater value on health practices in general.^53–55^ Healthcare professionals and oncology services can be made aware of possible differences in CIH disclosure behaviors and preferences between men and women when planning for their care.

Patient–care provider communication on CIH use was lacking among the study participants. Recent professional efforts aimed at improving integrative oncology services provide an opportunity to address such gaps in healthcare service.^56,57^ Leading cancer institutes incorporate CIH and supportive care information via their websites for patient and family education.^56,57^ This is important because cancer patients and survivors often do not communicate about their current CIH practices due to fear and anticipation of negative responses from a physician, a perception that CIH is irrelevant to the conventional medical treatment, and a sense that the medical professional is unwilling to contribute useful information about CIH practices.^58,59^ Poor communication about CIH use in this manner has the potential to create several medical care problems, such as supplement-drug interactions.^60–62^ Improved provider-initiated communication about CIH has implications for reducing such errors and addressing the varied needs of minority patients. As specific CIH products and techniques gain scientific backing or are found to be ineffective, healthcare providers will likely serve as the major channel of monitoring, communication, and referral for correct CIH use.^5,63,64^

The primary source of CIH information among Hispanic patients with CRC in this study was family or friends. These individuals have a major influence on the cancer patient's navigation of information and decision making^64^ and might be considered for inclusion in health communication messages so that they can make evidence-based recommendations that do not increase health risks in the survivor. Television, radio, and Internet were other major sources of CIH information for study participants.^65^ Increasingly, patients with cancer are relying on mass media messages for health and wellness recommendations; however, the quality and validity of the CIH information from these sources can be conflicting and unfounded.^57,65–67^ From a public health standpoint, these commonly accessed communication channels can be used to relay effective health messages about CIH use and safety.

The results reflected in this report have some limitations. The study used a single questionnaire as part of an ongoing population-based study. Responses depended on participants' recall of CIH use in the past 12 months, as well as their willingness to report their use accurately. The CIH items used failed to capture some culturally specific traditional practices, such as visiting a *curandero* or healer, and the language developed by the NIH for the CIH survey items might not be commensurate with cultural terms used to describe similar practices. Considering these limitations, this study provided one of the first descriptions of CIH use among a representative sample of Hispanics at various stages of CRC disease and survivorship. Future possible lines of investigation include discerning the reasons for lack of communication about CIH use between the study population and healthcare providers, as well as developing approaches to overcome barriers to improve communication in cancer care.

In conclusion, this study provides descriptive information about the use of CIH among Hispanic adults with a CRC diagnosis. Findings indicate that CIH use is common in this population, especially among women, but patient–provider communication about such practices is greatly lacking, presumably because of patient misperceptions surrounding conventional medicine practices. Understanding the CIH preferences and practices of underrepresented minority populations can improve communication between providers and patients as well as assist in the creation of culturally adapted health communication messages intended for cancer survivors.
